# 1243. Hepatitis D Virus and Hepatitis B Virus Epidemiology in Utah

**DOI:** 10.1093/ofid/ofac492.1074

**Published:** 2022-12-15

**Authors:** Matthew Hesterman, Braden Fallon, Paul Wilson, Melodie L Weller

**Affiliations:** University of Utah, School of Dentistry, Salt Lake City, Utah; University of Utah, School of Dentistry, Salt Lake City, Utah; University of Utah, School of Dentistry, Salt Lake City, Utah; University of Utah, School of Dentistry, Salt Lake City, Utah

## Abstract

**Background:**

Hepatitis Delta Virus (HDV) is a rare infectious disease that requires a helper virus (ex. Hepatitis B Virus (HBV)) for transmission. Worldwide, it is estimated that 12-72 million individuals are infected with HDV. Within the United States, an accurate measure of HDV prevalence is attenuated by limited testing and variable notifiable disease status. Recent reports have noted a significant shift in the international HDV epidemiology. This retrospective study was designed to further evaluate the prevalence and clinical features of HDV and HBV within the Utahn patient population.

**Methods:**

Within University of Utah Health, patient demographics, diagnostic codes (ICD9, ICD10) for HBV and HDV, CPT test codes and lab results were evaluated from 2000-2020. Univariate and multivariate analyses were performed. Timeseries analyses, including Mann-Kendall (MK) trend test, Bayesian Information Criterion (BIC) and Autoregressive Integrated Moving Average (ARIMA) were performed to characterize trends in HDV and HBV prevalence.

**Results:**

Between 2000-2020, 2878 HBV and 180 HDV patients were identified within the University of Utah Health system. The median age of the HBV and HDV patients was 45 years with 56% males and 42 years with 60% males, respectively. 10% of all HBV-tested patients were tested for HDV. The positivity rate of patients tested for HDV was 7%. Statistical analysis of race and ethnicity showed a significant difference in incidence and testing rates among the Utahn Asian population when compared against non-Asian populations. A significant increasing trend in the incidence of both HBV (MK=156, p=2.8e-6) and HDV (MK=83, p=0.01) was observed between 2000-2020. Within the timeframe analyzed, two structural breaks were observed for HDV/HBV incidence ratio.

**Conclusion:**

Together, our analysis suggests a significant change in the incidence of HDV and, due to the global temporal trend observed, may be suggestive of a change in HDV transmission pattern. Active surveillance of HDV in the United States and worldwide is warranted to further define these observed changes in HDV incidence.

**Disclosures:**

**All Authors**: No reported disclosures.

